# What role does PDL1 play in EMT changes in tumors and fibrosis?

**DOI:** 10.3389/fimmu.2023.1226038

**Published:** 2023-08-15

**Authors:** Yun-Chao Zhang, Yu-Ting Zhang, Yi Wang, Ya Zhao, Li-Jie He

**Affiliations:** ^1^ Department of Nephrology, Xi Jing Hospital, The Fourth Military Medical University, Xi’an, China; ^2^ Department of Medical Microbiology and Parasitology, Fourth Military Medical University, Xi’an, China

**Keywords:** epithelial-mesenchymal transformation, programmed death-ligand 1, immune escape, fibrosis, EMT-related disease

## Abstract

Epithelial-mesenchymal transformation (EMT) plays a pivotal role in embryonic development, tissue fibrosis, repair, and tumor invasiveness. Emerging studies have highlighted the close association between EMT and immune checkpoint molecules, particularly programmed cell death ligand 1 (PDL1). PDL1 exerts its influence on EMT through bidirectional regulation. EMT-associated factors, such as YB1, enhance PDL1 expression by directly binding to its promoter. Conversely, PDL1 signaling triggers downstream pathways like PI3K/AKT and MAPK, promoting EMT and facilitating cancer cell migration and invasion. Targeting PDL1 holds promise as a therapeutic strategy for EMT-related diseases, including cancer and fibrosis. Indeed, PDL1 inhibitors, such as pembrolizumab and nivolumab, have shown promising results in clinical trials for various cancers. Recent research has also indicated their potential benefit in fibrosis treatment in reducing fibroblast activation and extracellular matrix deposition, thereby addressing fibrosis. In this review, we examine the multifaceted role of PDL1 in immunomodulation, growth, and fibrosis promotion. We discuss the challenges, mechanisms, and clinical observations related to PDL1, including the limitations of the PD1/PDL1 axis in treatment and PD1-independent intrinsic PDL1 signaling. Our study highlights the dynamic changes in PDL1 expression during the EMT process across various tumor types. Through interplay between PDL1 and EMT, we uncover co-directional alterations, regulatory pathways, and diverse changes resulting from PDL1 intervention in oncology. Additionally, our findings emphasize the dual role of PDL1 in promoting fibrosis and modulating immune responses across multiple diseases, with potential implications for therapeutic approaches. We particularly investigate the therapeutic potential of targeting PDL1 in type II EMT fibrosis: strike balance between fibrosis modulation and immune response regulation. This analysis provides valuable insights into the multifaceted functions of PDL1 and contributes to our understanding of its complex mechanisms and therapeutic implications.

## 1 Introduction

The occurrence and development of EMT and mesenchymal-epithelial transformation (MET) can be divided into three biological subtypes in current research (1): Type I, which is primarily involved in the formation and development of embryos (2); Type II, which is primarily associated with tissue repair, regeneration, and fibrosis (3, 4); and Type III, which is linked to tumor invasion and immune suppression (5).

PDL1 has a crucial role in maintaining immune homeostasis and self-tolerance by inhibiting T cell immune response via binding to PD1 on T cells (6). However, recent studies have shown that PDL1 has other functions beyond its role in regulating immunity, including its interaction with EMT in the tumor microenvironment. The expression of PDL1 can increase during EMT, which induces immunosuppression and escape, and promotes cancer metastasis (7).

EMT represents a complex and intricate cellular process that extends beyond a simple transition from an epithelial to a mesenchymal state. During this process, cells acquire one or more hybrid epithelial/mesenchymal (E/M) phenotypes. Importantly, recent evidence indicates the existence of hybrid E/M phenotype cells characterized by elevated PDL1 expression (8, 9). These cells, with their hybrid E/M phenotype, may utilize increased PDL1 expression as a strategy to evade immune surveillance during tumor progression and metastasis. Consequently, this can confer resistance to therapeutic interventions. A deeper understanding of EMT and its correlation with elevated PDL1 expression not only enhances our comprehension of tumor development and immune evasion mechanisms but also paves the way for novel therapeutic strategies and targets.

Recent studies on EMT subtype III have revealed a significant correlation between EMT and immune activation, with high expression levels of immune checkpoint molecules such as PD1, PDL1, CTLA4, OX40L, and PDL2 observed in tissues with elevated EMT levels (10). Furthermore, interfering with the PDL1 pathway can impact the development of the EMT process within the tumor microenvironment (TME) (5, 11). While the relationship between EMT and immune checkpoints, including PDL1, has been extensively studied in the context of cancer, there is still much to be learned about their interaction in other areas of biology. However, some studies have suggested that EMT status could be a combined biomarker of PDL1, and that the two may have a bidirectional regulatory relationship beyond cancer (5). Further research is needed to fully understand the molecular mechanisms and potential clinical applications of this relationship.

EMT is a widespread phenomenon in the human body, and interrupting its progression can have a profound impact on the onset and progression of related diseases, particularly fibrosis in the context of EMT-II. Hence, reversing EMT has emerged as a crucial therapeutic strategy for combating fibrosis (2). The bidirectional interplay between EMT and PDL1 has given rise to a novel concept. Drawing from their mutual regulation in oncology, experts have suggested the existence of similar mechanisms in the context of fibrosis, such as the amplification of PDL1 to facilitate pulmonary fibrosis by hindering vimentin degradation, and the upregulation of PDL1 via Golm1 in the setting of liver fibrosis (12, 13).

Aside from the aforementioned types of fibrosis, there are numerous other fibrotic conditions where EMT plays a critical role, including peritoneal fibrosis (PF) triggered by peritoneal dialysis (PD). PD is a vital therapy for end-stage renal disease and has gained widespread acceptance in clinical settings (3). Long-term PD therapy can lead to decreased peritoneal function due to PF, which is a significant complication and the primary cause of long-term ultrafiltration failure in PD patients (14). During this pathological process, the peritoneum remains in a state of chronic inflammation, where EMT and its intermediate state induced by transforming growth factor-β (TGF-β) serve as major driving forces in the development of peritoneal fibrosis and subsequent functional deterioration (4, 15, 16). At the same time, TGF-β pathway drives the generation of congenital PD1 resistance characteristics and promotes the upregulation of PDL1 (17–20). Building upon the aforementioned phenomena, this review suggests a potential correlation between EMT and PDL1 within the realm of PF.

The primary objective of this review is to provide a comprehensive overview of the physiological roles of EMT and PDL1, while emphasizing recent advancements in understanding their reciprocal regulatory mechanisms in oncology. This includes examining the influence of EMT on PDL1 expression and the effects of inhibiting the PDL1 pathway on EMT progression. Additionally, this review aims to explore the interplay between EMT and PDL1 in non-tumor settings, with a specific focus on their implications in fibrosis.

In conclusion, EMT and its association with PDL1 have emerged as critical factors in various biological processes, including tumor progression, immune evasion, and fibrosis. Understanding the intricate relationship between EMT and PDL1 not only enhances our knowledge of disease mechanisms but also opens up new avenues for therapeutic interventions. By exploring the bidirectional interplay between EMT and PDL1, this review sheds light on their significance in both cancer and fibrosis contexts, with potential implications for the development of targeted therapies. Further research is warranted to unravel the underlying molecular mechanisms and explore the clinical applications of this dynamic interaction.

## 2 Challenges and opportunities in the application of PDL1

The discovery and clinical application of immune checkpoint inhibitors (ICIs) targeting PD1 and PDL1 were recognized by the 2018 Nobel Prize in Medicine as a major breakthrough in cancer treatment. This monumental achievement has brought about a paradigm shift in the approach to combating cancer, with immune checkpoint modulation taking center stage (21). At the heart of this intricate network lies the PD1/PDL1 pathway, a pivotal axis that exerts critical control over the T cell response, profoundly impacting their ability to recognize and eliminate malignant cells, including cancer cells and those infected with viruses (22). PDL1, a versatile molecule, not only adorns the surface of tumor cells and immune cells but also assumes an extracellular role, contributing to the intricate web of immunoregulation (23). Among these extracellular manifestations, exosomes and shed vesicles in the extracellular supernatant harbor PDL1, serving as an additional layer of regulation that complements the membrane-bound PD1/PDL1 axis (24).

### 2.1 Impact of PDL1 on patient survival in specific cancer subtypes: exploring clinical observations

Approaching the subject from a clinical standpoint, investigating the influence of PDL1 on patient survival in specific cancer subtypes provides profound insights into the mechanisms of tumor immune evasion and facilitates the development of personalized treatment strategies. Recent research has unveiled a positive correlation between heightened PDL1 activity, immune checkpoint markers, and elevated characteristics of partial EMT and glycolysis in various cancers. These findings suggest a close association between PDL1 and EMT in cancer progression, potentially synergistically fueling invasive tumor growth (8, 25).

Moreover, analyses encompassing multiple cancer types reveal that the co-enrichment of glycolysis and PDL1 is linked to shorter overall survival, compared to cases with PDL1 enrichment alone. Notably, investigations focused on colorectal cancer demonstrate a favorable prognosis associated with elevated PDL1 expression, underscoring the potential significance of PDL1 as a crucial prognostic factor and guiding personalized therapies (26).

In addition, studies conducted in high-grade chondrosarcoma (CS), retroperitoneal liposarcoma (LS), and undifferentiated pleomorphic sarcoma (UPS) highlight the immunoreactivity of PDL1. Particularly, patients with higher PDL1 immunoreactivity (>50%) within the tumor cells may form a rational cohort for exploring potentially beneficial PDL1/PD1 targeted therapies. These findings offer valuable insights for future in-depth investigations into PDL1 as a therapeutic target within specific sarcoma subtypes (27).

### 2.2 Challenges in clinical treatment: application limitations of PD1/PDL1 axis

The PD1/PDL1 axis serves as the arbiter of immune homeostasis. As T cell activity heightens, PD1 expression on the cell surface is elevated, playing a pivotal role in modulating the intensity of T cell response (28). While blocking the PD1/PDL1 axis can enhance the immune response against abnormal cells (29), excessive intervention may give rise to immune-related adverse events (irAEs) that can unpredictably affect any organ system. These irAEs encompass different aspects: firstly, there is an elevated susceptibility to lupus-like autoimmune diseases due to PD1 deficiency (30); secondly, high PDL1 expression can result in altered T cell development (31); and thirdly, extensive clinical intervention can lead to impaired organ function, such as thyroid dysfunction (hypothyroidism or thyrotoxicosis), pituitary inflammation (hypophysitis), adrenal insufficiency, pancreatic damage (diabetes), and other endocrine diseases resulting from extensive clinical intervention (32). Due to the intricacies of immune homeostasis in the human body and the unclear mechanisms of PD1/PDL1 regulation, the highly acclaimed PD1 and PDL1 inhibitors face two major challenges in clinical treatment: limitations in application and significant side effects.

### 2.3 PD1-independent intrinsic PDL1 signaling

Most studies have focused on PDL1’s role as a ligand of PD1 and its effect on PD1-expressing T cells. However, recent research has revealed the broader significance of cancer cell-intrinsic PDL1 signaling in controlling various aspects of tumor growth, immune effects, DNA damage response, and gene expression regulation, many of which operate independently of PD1 (33). These effects include the following: firstly, PDL1 can interact with CD80, transmitting inhibitory signals to activated T cells (34); secondly, PDL1-targeted therapy can reduce mTOR activity and glycolysis in tumor cells, even in the absence of T cells (35); thirdly, in the absence of PD1, PDL1 serves as a shield for tumor cells against cytotoxicity induced by type I and II interferons and cytotoxic T lymphocytes (36); fourthly, PDL1 can regulate extracellular matrix (ECM) components and their proteolytic remodeling products, promoting immune escape and accelerated invasion of tumor cells (37); and lastly, PDL1 and TGF-β form complementary signaling pathways that promote tumor fibrosis and epithelial transformation to stroma (38).

### 2.4 The intricate relationship of EMT and PDL1: immune evasion and fibrotic pathologies

**Figure 1:**
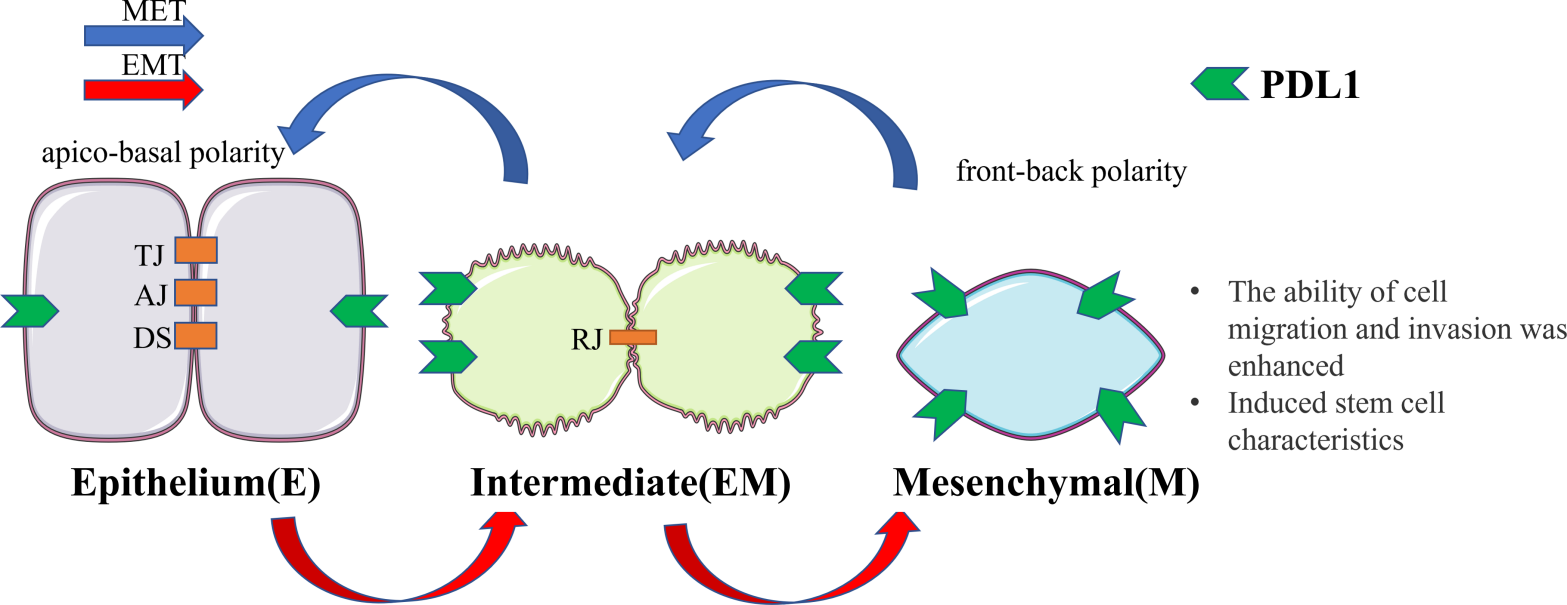
Cellular changes during EMT and MET. Upon undergoing EMT, cellular adhesion is diminished, leading to the loss of the epithelial phenotype and the gradual acquisition of a mesenchymal phenotype. In this transitional state, there is an upregulation of PDL1 expression. This transformation endows cells with the capacity for distant metastasis and redifferentiation. Conversely, MET induces the reversal of these alterations. EMT, epithelial-mesenchymal transformation; MET, mesenchymal - epithelial transformation; TJ, tight junction; AJ, adhesive junctions; DS, desmosome; RJ, residual junction.

Based on the aforementioned studies regarding the impact of PDL1, its intrinsic effects, suggest that changes in EMT occur during the production of PDL1 (7, 39). When coupled with the examples highlighted in the introduction indicating that the relationship between EMT and PDL1 is widely prevalent in immune escape of tumors and organ fibrosis (5, 10–13), it is reasonable to conclude that EMT is closely linked to PDL1, and the signaling pathway between EMT and PDL1 may exist in the major physiological subtypes of EMT (**Figure 1**).

## 3 Exploring the impact of EMT on PDL1 expression: dynamic changes in tumor biology

### 3.1 Concordant changes of PDL1 expression during the EMT process

Currently, numerous studies have demonstrated a strong correlation between the state of EMT and the expression of immune checkpoint molecules, particularly PDL1, which exhibits dynamic changes as EMT progresses (10). This view is further supported by comparing the contents of EMT-related DNA, RNA, and protein in different tumor types: firstly, in lung cancer, a positive correlation is observed between the expression of EMT markers, such as Snail and Vimentin, and the levels of PDL1 (6). Conversely, the expression of E-cadherin, a marker of epithelial characteristics, shows a negatively correlated with PDL1 expression (40); secondly, in breast cancer, mesenchymal cancer cell lines demonstrate a higher proportion of PDL1-positive cells. Activation of ZEB1 or Snail leads to an increase in PDL1 expression (41); thirdly, in patients with cholangiocarcinoma, high expression of EMT markers, such as ZEB1, N-cadherin, and Vimentin, along with low expression of E-cadherin, are associated with increased PDL1 expression (42); lastly, similar results are observed in esophageal squamous cell carcinoma, patients with elevated levels of EMT marker ZEB1 and positive PDL1 have the worst prognosis (43). These findings collectively emphasize the close relationship between EMT and PDL1 expression across various cancer types.

### 3.2 Unraveling the complexities: mechanisms of PDL1 expression in the EMT process

Extensive literature has consistently indicated that in the tumor microenvironment, as EMT progresses, there is a notable upregulation of PDL1 expression (5, 10, 11, 40–45). Many scholars have put forward their own views on the underlying mechanisms governing PDL1 expression during EMT progression: firstly, TGF-β, the primary inducer of EMT, boosts the expression of PDL1 via the PI3K/AKT and MEK/ERK pathways in breast cancer. It is suggested that the EMT process itself, rather than the direct influence of TGF-β, leads to the heightened expression of PDL1 (46); secondly, interference with ZEB1 at the RNA level can bring about a reduction in PDL1 mRNA and protein levels in esophageal squamous cells (43). Similar results were obtained in non-small cell lung cancer, indicating that the expression of PDL1 is regulated by the miR-200/ZEB1 channels and that there exists a miR-200 binding site on PDL1 (47). Based on this, some scholars have proposed a clear correlation between the heightened expression of PDL1 and the augmentation of ZEB1 and ZEB2, along with the reduction of miR200a and miR200c. ZEB1, which functions as an EMT activator and a transcriptional inhibitor of miR-200, can alleviate the inhibitory effect of miR-200 on PDL1 (48, 49); thirdly, PDL1 is enriched in tumor stem cells through the EMT/β-catenin/STT3/PDL1 signaling axis, in which EMT induces N-glycosyltransferase STT3 via β-catenin transcription, and then stabilizes PDL1 and upregulates (50); fourthly, EMT transcription factors, including the ZEB, Twist, and Snail family proteins, have also been shown to regulate PDL1 expression through the MAPK pathway (42, 51, 52); lastly, there are alternative viewpoints: the downregulation of E-cadherin by shRNA can reduce PDL1 expression (53). Knockout of Snail can significantly inhibit EMT but has no effect on PDL1 expression. Knockout of c-Myc suppresses PDL1 expression while not affecting EMT (54). In summary, the precise mechanisms governing PDL1 expression during the EMT process warrant further investigation.

## 4 Divergent changes in EMT following PDL1 intervention in oncology

### 4.1 Co-direction changes of EMT after intervention of PDL1 expression

Numerous studies have shown the therapeutic potential of PDL1 in reducing EMT and inhibiting cell invasion in tumors (11, 55–57). High expression of PDL1 plays an important role in maintaining EMT status in various types of cancer, such as breast cancer, hepatocellular carcinoma, esophageal carcinoma, and renal cell carcinoma (58, 59). Additionally, extracellular PDL1 has similar biological effects (57, 60). Corresponding to the changes observed in PDL1 during the EMT process, interventions targeting PDL1 expression also induce EMT alterations in various types of tumors. For example: firstly, in lung cancer, the upregulation of PDL1 expression by ATM through the JAK1, 2/STAT3 signaling pathway promotes EMT and tumor metastasis, while the inhibition of ATM reduces PDL1 expression via the JAK/STAT3 signaling pathway, resulting in diminished EMT and suppressed tumor metastasis in mouse models (11); secondly, in head and neck squamous cell carcinoma, the loss of CMTM6, a regulator of PDL1 expression, inhibits PDL1 expression and leads to low EMT expression. Additionally, a positive correlation between the expression of CMTM6 and EMT-related genes has been found in TCGA. The authors propose that CMTM6 modulates the EMT process in cancer through the Wnt/β-catenin signaling pathway (55); thirdly, in esophageal cancer cells, PDL1 expression significantly promotes EMT phenotype, with the cytoplasmic domain of PDL1 plays an important role in this process (58); fourthly, in renal cell carcinoma, downregulation of PDL1 by lentivirus can inhibit the expression of EMT markers such as Vimentin, while upregulation of PDL1 can promote the expression of these EMT markers by activating SREBP-1 (59); lastly, in non-small cell lung cancer, downregulation of Circ-CPA4 suppresses the expression of PDL1 through let-7miRNA, thereby affecting the activity of T cells, and then the process of EMT is changed. Additionally, the paper proposes an intriguing phenomenon: upregulation of Circ-CPA4 enables non-small cell lung cancer cells to bind to CD8+ T cells in the way of secreting PDL1 exosomes, thereby effectively deactivating them (57). This complementary function further enhances the effect of PDL1.

### 4.2 Shared regulatory signaling of EMT and PDL1

**Figure 2:**
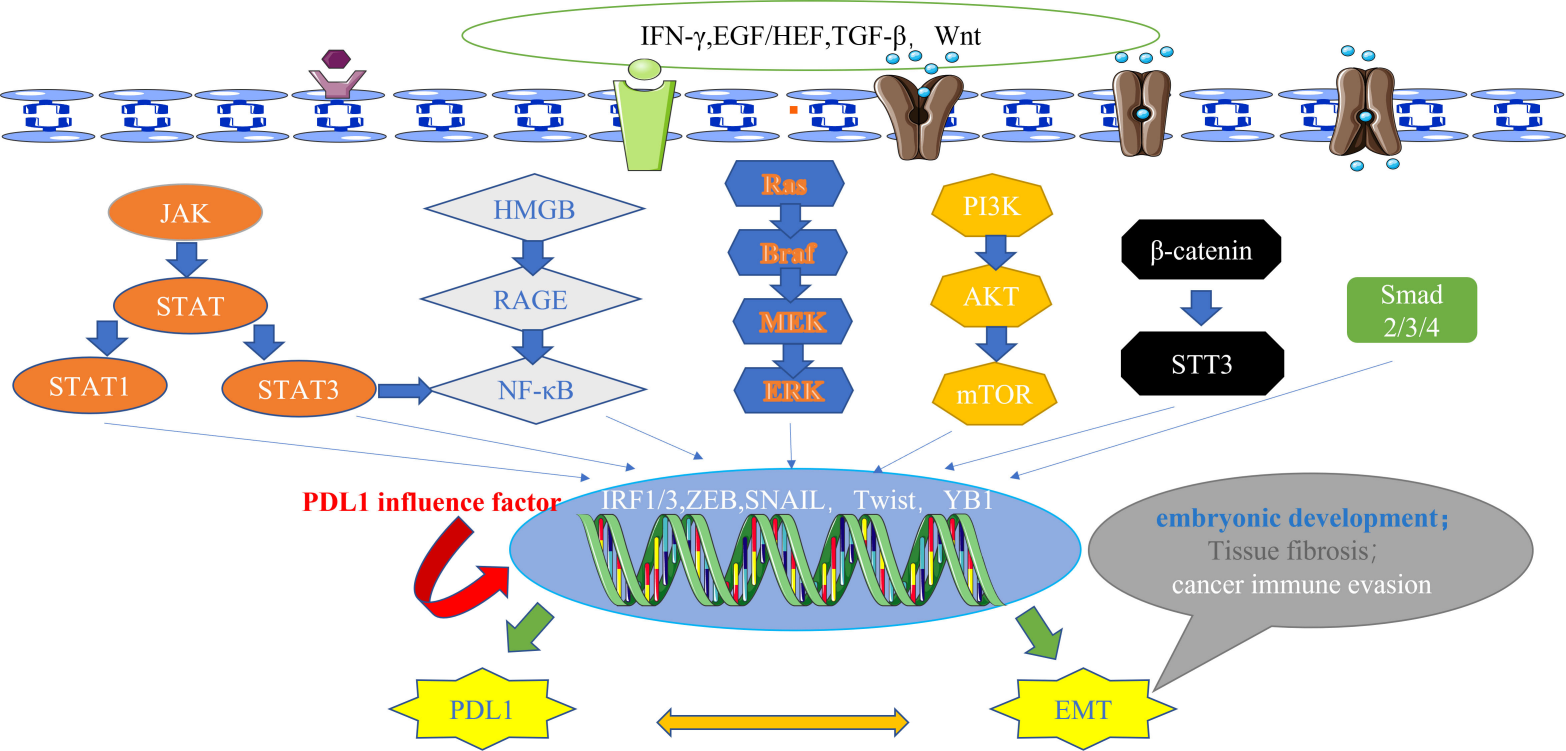
The main signal pathways that affect the expression of PDL1.These pathways also have downstream signals, including ZEB and Snail, which act as transcription factors for EMT and regulate the expression of PDL1, highlighting the bidirectional regulatory relationship between EMT and PDL1 at the signaling pathway level. IFN-γ, interferon-γ; EGF, epidermal growth factor; HGF, hepatocyte growth factor; TGF-β, transforming growth factor–β; Wnt, wingless; JAK, janus kinase; STAT, signal transducer and activator of transcription; HMGB1, high mobility group protein 1; RAGE, receptor for advanced glycation end products; Ras, rasopathes; Braf, RAF family serine/threonine protein kinases; MEK, MAPK kinase; ERK, extracellular-signal regulated protein kinase; PI3K, phosphoinositide 3-kinase; AKT, protein kinase B; mTOR, mammalian target of rapamycin; STT3, N-oligosaccharide transferase complex; Smad, skin growth factor; IRF, interferon regulatory factor; ZEB, zinc finger E-box binding homeobox; PDL1, programmed death ligand 1; EMT, epithelial-mesenchymal transformation.

Currently, a wealth of evidence supports the existence of multiple signaling pathways that intricately regulate PDL1 expression. These pathways encompass a spectrum of influential factors, which can be broadly categorized into six main types: firstly, JAK/STAT pathway (61, 62); secondly, NF-κB pathway (63, 64); thirdly, MAPK pathway (65); fourthly, PI3K/AKT/mTOR pathway (46, 66); fifthly, TGF-β/Smad pathway (60); and lastly, β-catenin/STT3 pathway (50). Remarkably, EMT marker molecules such as ZEB1 and Snail have been implicated in these six signaling cascades, further supporting the reciprocal relationship between EMT and PDL1 at the signaling pathway level (62) (**Figure 2**).

**Figure 3:**
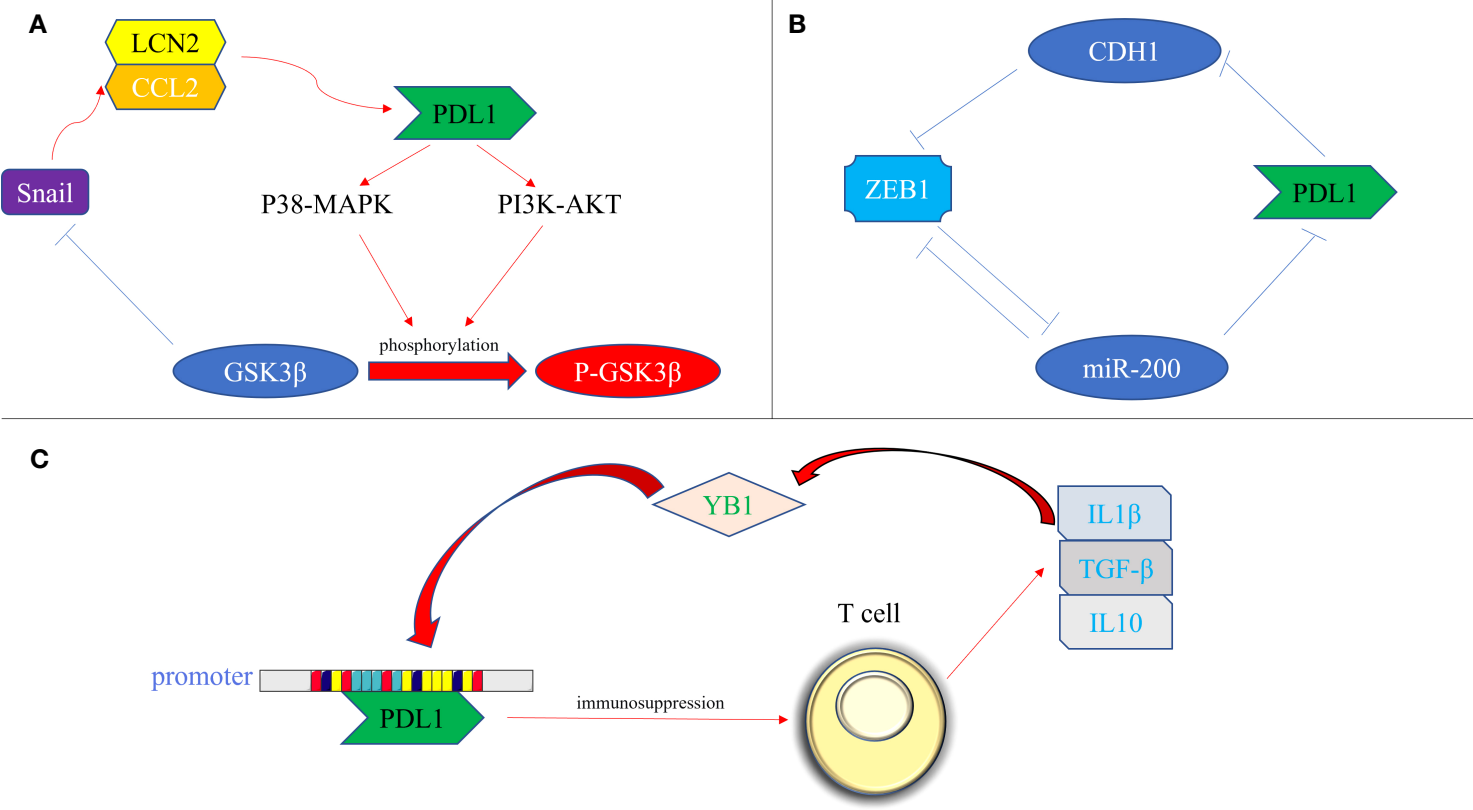
Bidirectional regulation of EMT transcription factors and PDL1. **(A)** At the protein level. PDL1 activates the p38-MAPK or AKT pathways, leading to the phosphorylation of GSK3β and subsequent upregulation of Snail expression. Snail, in turn, promotes the generation of the CCL2/LCN2 complex, inducing an immunosuppressive state in cells and further enhancing PDL1 expression; **(B)** At the miRNA level, ZEB1 acts as a transcriptional repressor of miR-200, alleviating the inhibitory effect of miR-200 on PDL1 expression. Additionally, PDL1 can influence ZEB1 through the generation of CDH1; **(C)** At the transcriptional level, YB1 directly binds to the promoter sequence of PDL1, promoting PDL1 production. The excessive presence of PDL1 induces the release of fibrosis-promoting factors, such as TGF-β, from T cells, thereby promoting the upregulation of YB1. PDL1, programmed death ligand 1; P38-MAPK, p38 mitogen activated protein kinase; PI3K, phosphoinositide 3-kinase; AKT, protein kinase B; GSK3β, glycogen synthase kinase 3β; P- GSK3β, phosphorylated glycogen synthetase kinase 3β; CCL2, C-C motif chemokine ligand 2; LCN2, lipocalin 2; ZEB, zinc finger E-box binding homeobox; miRNA-200, micro RNA 200 family; YB1, Y-box binding protein 1; IL1β, interleukin 1; IL10, interleukin 10; TGF-β, transforming growth factor–β.

The transcription factors involved in promoting tissue EMT transformation contribute to the expression of PDL1. Moreover, PDL1 itself exerts an influence on the secretion of EMT transcription factors, which can be classified into the following three types (**Figure 3**): firstly, at the protein level, an intricate interplay between Snail and PDL1 has been observed. PDL1 has the ability to induce the phosphorylation of GSK3β through activation of the p38-MAPK or AKT pathways, ultimately promoting the expression of Snail (where GSK3β serves as a ubiquitin ligase for Snail) (67). Furthermore, the generation of the CCL2/LCN2 complex by Snail can induce cells to adopt an immunosuppressive state, leading to the upregulation of PDL1 expression (68); secondly, at the miRNA level, the impact of ZEB1 on PDL1 is notable. The miR-200 family acts as a targeted inhibitory signal for PDL1, while ZEB1 acts as both an EMT activator signal and a transcriptional suppressor for miR-200, thereby relieving the inhibitory effect of miR-200 on PDL1 expression (49). In turn, PDL1 can influence ZEB1 through the generation of CDH1 (69); thirdly, at the transcriptional level, YB1 participates in the regulation of PDL1. YB1 directly binds to the promoter sequence of PDL1, thereby inducing the production of PDL1. Excessive PDL1, in turn, triggers the release of fibrosis-promoting factors by T cells, such as TGF-β, thereby promoting the upregulation of YB1 (70).

### 4.3 Unique scenario: accelerated EMT transformation following downregulation of PDL1

Contrary to the anticipated alignment between EMT and PDL1 alterations discussed earlier, divergent alterations in EMT may occur after intervention in PDL1. In current therapeutic research of PDL1, a surprising revelation has emerged: high PDL1 expression does not ensure a favorable response to PD1 inhibitors, while low or even absent PDL1 expression does not necessarily signify an unresponsive state to PD1/PDL1 antibodies (71, 72). Moreover, the influence of PDL1 on EMT is highly dependent on the stage of the disease, showcasing intricate ramifications as the disease unfolds. Primarily, the reduction of PDL1 levels disrupts the binding of PD1/PDL1, effectively impeding macrophage apoptosis and mitigating the detrimental consequences of inflammation. Consequently, the progression of EMT is influenced, leading to a reduction in the magnitude of tissue fibrosis (61). Notably, the downregulation of PDL1 following primary infection plays a pivotal role in restoring protective immunity. Nevertheless, this process entails the emergence of post-infection inflammation and the consequent progression of fibrosis (73).

## 5 The multifunctional role of PDL1 in EMT: from immune evasion to developmental promotion and fibrosis

Based on the strong correlation between EMT and PDL1 in the field of oncology, some scholars have carried out PDL1 studies on other subtypes of EMT, and obtained results to test the hypothesis that the mechanism linking EMT and PDL1 widely exists in the human body.

### 5.1 PDL1 in embryonic development of type I EMT

The physiological mechanism of EMT permeates through various facets of life, encompassing primordial cell differentiation, organ development, and even final death. Expanding upon the research conducted on PDL1 and its relationship with EMT in tumor contexts. Intriguingly, evidence has emerged to substantiate this notion: firstly, the inhibition of PDL1 has been shown to regulate the EMT process through ATF3 signaling, subsequently influencing angiogenesis and ultimately culminating in embryonic death (74); secondly, the blockade of PDL1 leads to the accumulation of T follicular regulatory cells within the uterine environment during the second trimester, provoking various immune dysfunctions and even triggering pregnancy termination (75); thirdly, PDL1 induces macrophages polarization towards the M2 phenotype during early pregnancy, facilitating the establishment of maternal-fetal tolerance (76); lastly, soluble PDL1 emerges as a pivotal factor in embryonic development during pregnancy. Trophoblast cells actively secrete soluble PDL1, thereby initiating the PD1/PDL1 pathway and orchestrating the M2 polarization of macrophages, crucial for sustaining a conducive microenvironment for embryonic growth (77).

The literature on PDL1 in embryonic development mainly focuses on its immune regulatory functions, such as its influence on M2 polarization of macrophages and pregnancy outcomes [M2 macrophages are closely related to the occurrence of EMT (78)]. Nonetheless, the precise mechanism underlying the intricate interplay between PDL1 and EMT during embryonic development remains elusive. While some evidence suggests potential roles for PDL1 beyond immune cell regulation, such as cell differentiation and tissue formation, these findings are currently limited. Further investigations are needed to unravel the intricate relationship between PDL1 and EMT during the journey of embryonic development.

### 5.2 PDL1 in tissue repair and fibrosis of type II EMT

Considering the well-documented impact of PDL1 on M2 macrophage polarization and the aforementioned instances of EMT and PDL1 in the field of fibrosis, scholars have extensively investigated the dynamics of PDL1 in fibrotic conditions, especially those arising from chronic inflammation. Within a chronic inflammatory microenvironment, the release of inflammatory mediators triggers an uncontrolled proliferation of fibroblasts, their conversion to an EMT phenotype, and ECM deposition, ultimately culminating in collagen overproduction and the onset of fibrosis. In this intricate cascade, the involvement of PDL1 may play a vital role (79, 80).

#### 5.2.1 The dual role of PDL1: immunomodulation and promoting fibrosis across fibrosis diseases

##### 5.2.1.1 The fibrotic role of PDL1 in idiopathic pulmonary fibrosis

Idiopathic pulmonary fibrosis (IPF) is a chronic, progressive, and fibrotic interstitial lung disease characterized by chronic epithelial injury and the regenerative failure of the alveolar compartment (81). Remarkably, IPF and lung cancer exhibit striking biomolecular similarities, with EMT emerging as a critical component in the pathogenesis of IPF fibrosis (82, 83).

**Figure 4:**
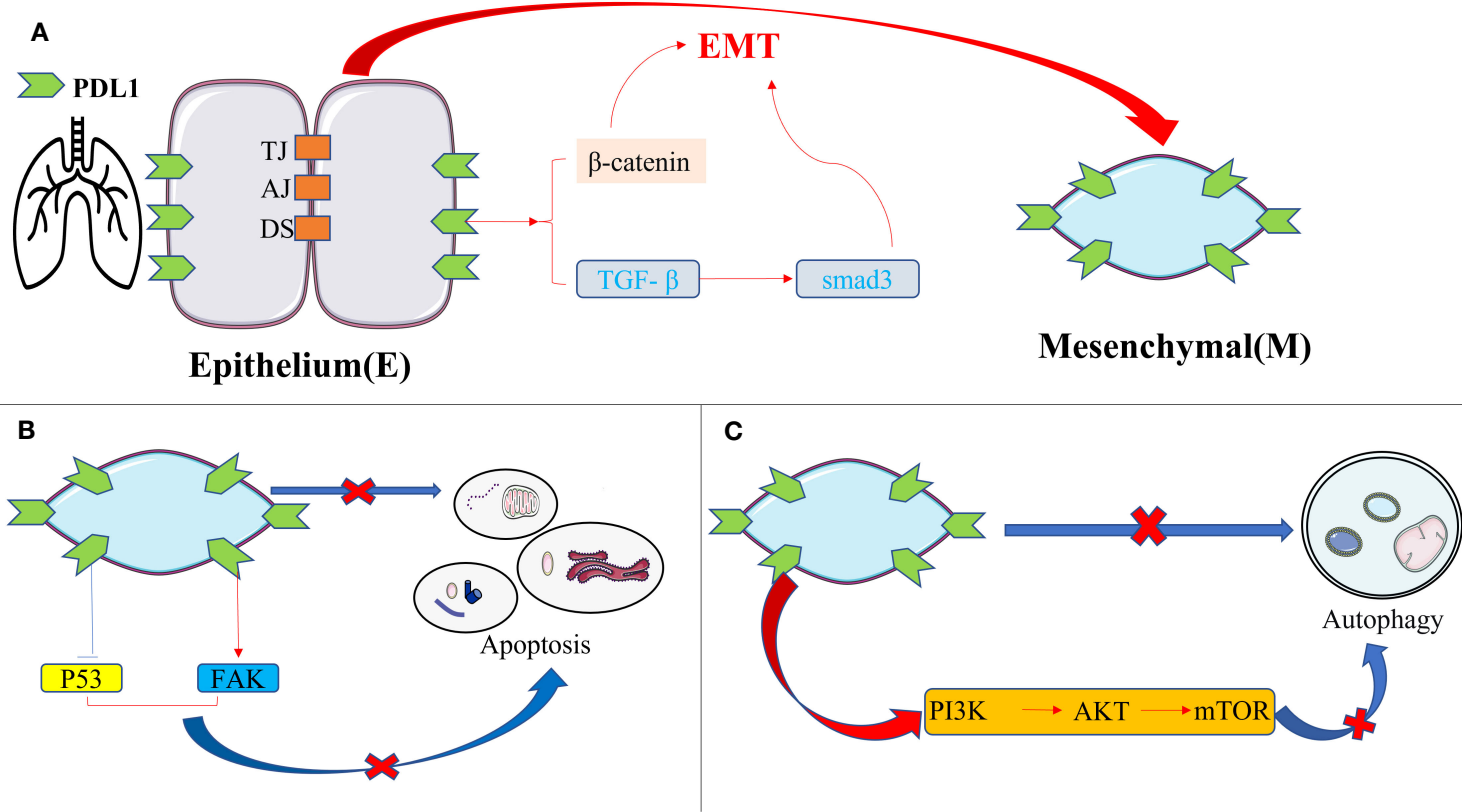
The intrinsic pro-fibrotic effect of PDL1 in pulmonary fibrosis. **(A)** PDL1 promotes fibrosis by mediating fibroblast-to-myofibroblast transition and EMT via TGF-β/Smad3 and β-catenin signaling pathways; **(B)** Overexpression of PDL1 leads to myoblasts’ resistance to apoptosis and escape from macrophage phagocytosis through inhibition of p53 pathway or activation of FAK pathway, ultimately leading to excessive proliferation of myoblasts and triggering IPF; **(C)** PDL1 can inhibit autophagy of fibrosis-related cells through the PI3K/AKT/mTOR signal pathway. PDL1, programmed death ligand 1; EMT, epithelial-mesenchymal transformation; TJ, tight junction; AJ, adhesive junctions; DS, desmosome; TGF-β, transforming growth factor–β; Smad, skin growth factor; FAK, focal adhesion kinase; PI3K, phosphoinositide 3-kinase; AKT, protein kinase B; mTOR, mammalian target of rapamycin.

Emerging literature highlights the potential of PDL1 inhibition in reducing fibrosis severity in pulmonary fibrosis, and the PDL1 pathway mainly plays a role in promoting fibrosis rather than its immunomodulatory role (84). The fibrotic effect of PDL1 in IPF arises from intricate cellular interactions and signaling pathways, with the following mechanisms assuming paramount significance(**Figure 4**): firstly, overexpression of PDL1 in lung fibroblasts exerts resistance to myoblast apoptosis and eludes macrophage phagocytosis via the inhibition of p53 pathway or activation of FAK pathway. Consequently, excessive myoblast proliferation ensues, instigating IPF development (85–87); secondly, PDL1 mediates the transition of pulmonary fibroblasts into myofibroblasts and instigates EMT induction via TGF- β, facilitated by the Smad3 and β-catenin signaling pathways, thereby promoting fibrosis (88, 89); thirdly, upregulation of PDL1 in pulmonary fibroblasts hampers autophagy-induced myofibroblast proliferation and ECM deposition, ultimately leading to pulmonary fibrosis. Overexpression of PDL1 can inhibit autophagy of fibrosis-related cells through the PI3K/AKT/mTOR signal pathway, up regulate TGF-β and fibrosis-related factor a-SMA (90–92); fourthly, elevated PDL1 expression in pulmonary fibroblasts impedes adaptive immunity, fostering pulmonary fibrosis. Notably, immune dysregulation has emerged as a key driver of IPF, with M2 macrophages, Th17 cells, CD8+T cells, and Tregs promoting fibrosis, while Th1 and TRM cells appear to exert protective effects (93). Furthermore, current evidence suggests that upregulation of PD1/PDL1 in IPF may prompt the differentiation of CD4+T cells into Tregs and lead to depletion of macrophages, thereby enabling myofibroblasts to evade immune clearance and resist macrophage-induced phagocytosis (94, 95).

These reports provide valuable insight into the fibrogenic role of PDL1 in IPF. It is noteworthy that the mechanism by which PDL1 operates in IPF mirrors the mutual regulation observed between PDL1 and EMT in the context of tumors. In the case of IPF, PDL1 may also impact EMT in the following ways: firstly, the PDL1 signaling pathway, including STAT3, p53, β-catenin, and PI3K/AKT/mTOR, may affect alveolar epithelial cell migration and EMT induction mediated by TGF-β (96–99); secondly, PDL1 in IPF can impede autophagy, a tumor suppressor process that selectively downregulates key transcription factors associated with EMT, thereby hindering metastasis in the early stages (100); lastly, PDL1 in IPF can induce Tregs and M2 macrophages. Tregs promote EMT in alveolar epithelial cells through β-catenin, while M2 macrophages foster myofibroblast activation and EMT, resulting in collagen deposition and upregulation of fibrogenic cytokines (101, 102). The above three points contribute to our comprehension of the role of PDL1 in IPF, shedding light on the potential interplay between PDL1 and EMT in this condition. Importantly, the latest research in 2023 further clarifies their correlation, as heightened PDL1 levels impede vimentin ubiquitination, consequently amplifying vimentin levels and intensifying the EMT of alveolar epithelial cells, thus exacerbating pulmonary fibrosis (12).

##### 5.2.1.2 immunomodulatory functions of PDL1 in liver fibrosis and its potential impact on EMT

**Figure 5:**
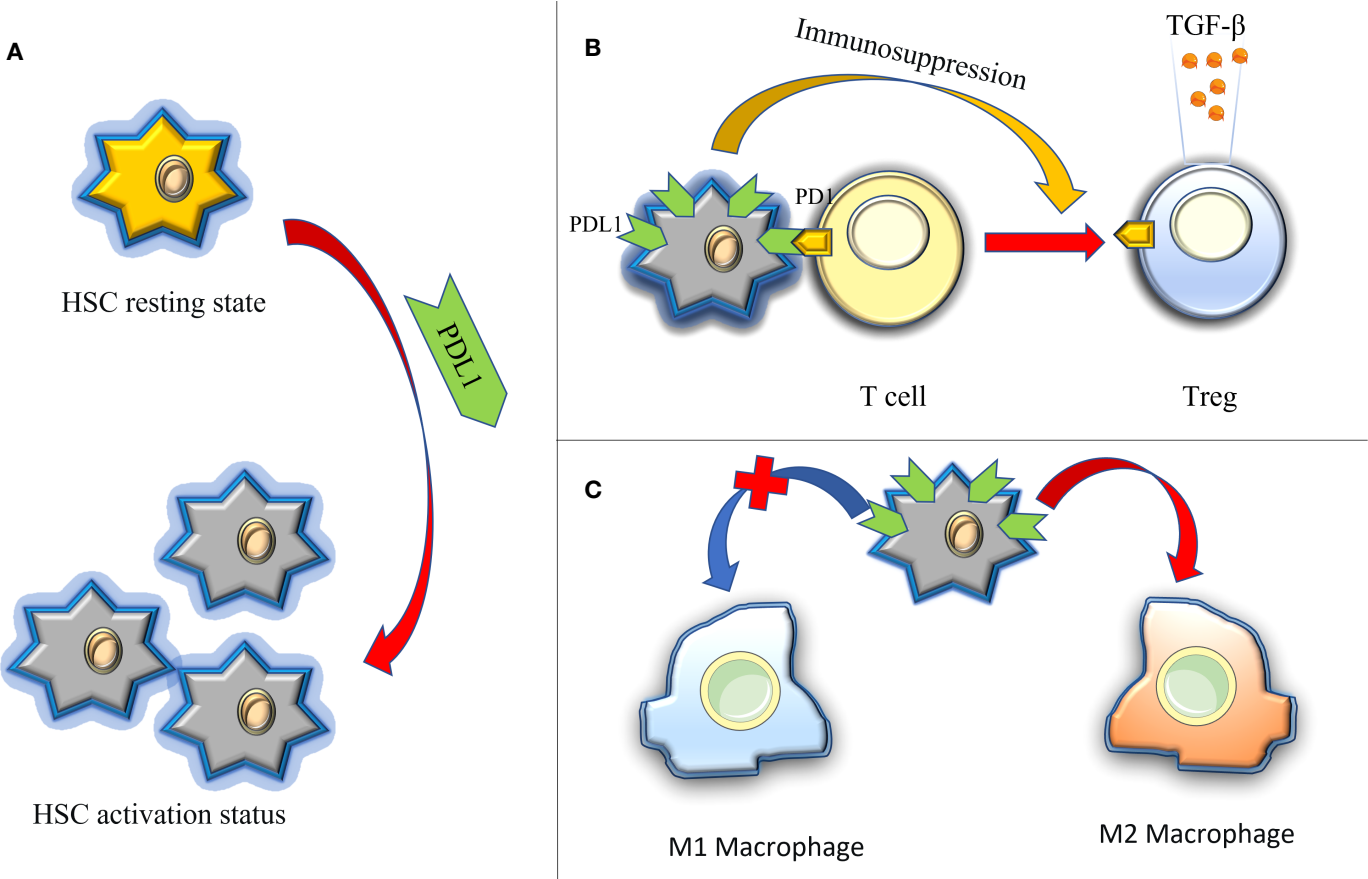
The immune regulation of PDL1 in hepatic fibrosis, thus promoting the role of fibrosis. **(A)** PDL1 activates HSCs (HSCs are considered to be the main effector cells of liver fibrosis); **(B)** PDL1 induces the production of Tregs and releases exocrine bodies showing TGF-β; **(C)** PDL1 stimulates the polarization of M2 macrophages. HSCs, hepatic stellate cells; PDL1, programmed death ligand 1; PD1, programmed cell death protein 1; TGF-β, transforming growth factor–β.

The liver, as a central immune organ in the human body, assumes a crucial role in maintaining overall immune function. Recent studies have shed light on immune dysregulation, particularly the PD1/PDL1 immune checkpoint disorder, as a significant cause of liver function damage and fibrosis (103, 104). In contrast to pulmonary fibrosis, the impact of PDL1 on liver fibrosis primarily stems from its immunomodulatory functions(**Figure 5**): firstly, in the context of hepatitis C, upregulated PDL1 in hepatocytes induces the generation of Tregs and follicular regulatory T cells, releasing exosomes rich in TGF-β. This inhibits the host T cell response to persistent infection, thereby promoting liver injury and fibrosis (105); Secondly, hepatic stellate cells (HSCs), are considered to be the main effector cells of liver fibrosis, drive immunosuppressive responses in homeostasis, such as Treg induction, T cell apoptosis, or suppression of cytotoxic CD8+ T cells (106). Current evidence suggests that the activation and augmentation of HSCs are closely associated with the expression of PDL1 (107); lastly, the contribution of M2 macrophages to liver fibrosis has gained substantial recognition, with PDL1 expression believed to promote the expansion of the M2 macrophage population within the microenvironment of liver fibrosis (108).

Liver fibrosis entails a pathological process in which hepatocytes, HSCs, and bile duct cells undergo EMT and transform into myofibroblasts, leading to excessive deposition of ECM and persistent liver injury (109). The mechanism of PDL1 in hepatic fibrosis shares similarities with pulmonary fibrosis and may also affect EMT in the liver in the following ways: firstly, PDL1 can induce the production of TGF- β in liver fibrosis. Consistent with pulmonary fibrosis, EMT signaling pathways involving TGF- β, such as Smad, PI3K/AKT, STAT3 and p53 pathways, also operate in hepatic fibrosis (110–113); secondly, PDL1 activates HSCs, resulting in the secretion of various substances, including ECM components, matrix metalloproteinases, tissue inhibitors of metalloproteinases, chemokines, growth factors, and TGF- β. The latter two exert autocrine or paracrine effects on HSCs and other cells, fostering cell proliferation, migration, and EMT transformation (114); lastly, PDL1 stimulates the polarization of M2 macrophages, which in turn downregulates the expression of E-cadherin and upregulates the expression of vimentin in hepatocytes, promoting EMT (115). While these three points offer valuable insights into the role of PDL1 in hepatic fibrosis, conclusive evidence linking PDL1 and EMT in this context remains elusive, underscoring the need for further investigation.

#### 5.2.2 The therapeutic potential of targeting PDL1 in type II EMT fibrosis: striking a balance between modulating fibrosis and regulating the immune response

Studies conducted on both individuals and mouse models of tissue fibrosis have shed light on the multifunctionality of the PDL1 axis in fibrosis, encompassing both fibrosis promotion and immunomodulation. Consequently, many preclinical investigations in mouse models have explored the potential of targeting the PD1/PDL1 axis for fibrosis treatment. Many of these studies have demonstrated that obstructing the PD1/PDL1 axis with PDL1 monoclonal antibodies can effectively reduce fibrosis severity in mice (87, 90, 95, 107, 116). However, it is important to note that not all PDL1 antagonistic therapies have yielded successful outcomes. For instance, despite human mesenchymal stem cells being utilized in IPF therapy for their immune regulatory properties, recent animal studies have revealed that administering PD1/PDL1 inhibitors can reverse their antifibrotic effect (117, 118).

The development of fibrosis stems from the intricate interplay between anti-fibrosis and pro-fibrosis mediators within a microenvironment comprised of diverse cell types (95). Research suggests that downregulating PDL1 in fibroblasts can alleviate pulmonary fibrosis through various mechanisms. However, it is important to consider that excessive activation of T cells resulting from this immune response may disrupt this delicate balance required for effective fibrosis modulation (119, 120). Thus, while targeting the PD1/PDL1 axis to reduce fibrosis holds promise as a novel therapeutic approach, extensive research is still needed to determine the optimal equilibrium between immune activation and inhibition of fibrosis.

#### 5.2.3 Potential link between EMT and PDL1 in peritoneal fibrosis: insights from pulmonary and hepatic fibrosis

In line with pulmonary and hepatic fibrosis, peritoneal fibrosis (PF) represents a fibrotic manifestation centered around the process of EMT. Presently, it is widely acknowledged that decreased peritoneal function induced by PF is a major complication of long-term peritoneal dialysis(PD) therapy and a primary cause of long-term ultrafiltration failure of PD (14). The pathological progression of PF bears similarities to that of pulmonary and hepatic fibrosis: firstly, EMT and its intermediate state, driven by the central TGF-β signaling pathway, exert a significant driving force in the genesis and subsequent functional deterioration of PF (4, 15, 16); secondly, M2 macrophages and Tregs may also represent critical components of PF, similar to their impact on pulmonary and hepatic fibrosis (121); thirdly, as observed in the lung and liver, PD1/PDL1 expression can be detected in the abdominal cavity (122, 123); fourthly, human peritoneal mesothelial cells (HPMCs), which are the primary cells involved in PF, are also implicated in various types of cancer and contribute to the maintenance of the tumor microenvironment. Notably, the phenotypic alterations observed in HPMCs during PF exhibit similarities to those witnessed in cancer (124). Considering these mechanisms, this review postulates a potential correlation between EMT and PDL1 in the field of PF.

## 6 Conclusion and prospects

Among the three subtypes of EMT, this review has focused on exploring the potential correlation between EMT and PDL1 in fibrosis, as well as the mechanism of immune escape in tumor environments. As mentioned above, EMT serves various physiological functions in the body, and the immune regulation mediated by PDL1 is a fundamental element for normal human survival. However, beyond the realm of tumors, the precise regulatory mechanisms connecting EMT and PDL1 remain elusive.

At present, the research on EMT and PDL1 primarily concentrates on tumor-related studies and membrane surface PDL1, which limits the breadth of investigation in other biological fields, such as embryonic development, wound repair, and fibrosis. An intriguing avenue to explore is the role of extracellular PDL1, which might exert physiological effects similar to PDL1 on the membrane surface. Exploring this aspect could enhance our understanding the EMT process and enable interventions in distant tissues. Furthermore, the existing mechanisms necessitate further exploration. Therefore, this review proposes several hypothetical scenarios in which the manipulation of PDL1 expression could regulate the biological effects of EMT, such as promoting the development of premature infants akin to hormone therapy, affecting the process of fibrosis caused by chronic inflammation to maintain the normal biological function of tissue, and examining the impact of extracellular PDL1 on EMT subtypes beyond tumors.

In conclusion, the intricate bidirectional regulation between EMT and PDL1 underscores the interconnectedness of these fields, as evidenced by shared responses like immune evasion in tumors and pro-fibrotic effects in fibrotic diseases. This interplay presents promising opportunities for the development of innovative therapeutic approaches to effectively manage EMT-associated diseases. However, to fully comprehend the underlying regulatory mechanisms and identify potential targets for therapeutic interventions, further comprehensive research is indispensable.

## Author contributions

YCZ and YTZ contributed to conception and design of the study. YCZ organized the database and wrote the first draft of the manuscript. YTZ and YW wrote sections of the manuscript. All authors contributed to manuscript revision, read, and approved the submitted version.
